# A Delayed Diagnosis of Autism Spectrum Disorder in the Setting of Complex Attention Deficit Hyperactivity Disorder

**DOI:** 10.7759/cureus.25825

**Published:** 2022-06-10

**Authors:** Michelle I Malwane, Eric B Nguyen, Sergio Trejo, Erica Y Kim, José R Cucalón-Calderón

**Affiliations:** 1 Pediatrics, University of Nevada Reno School of Medicine, Reno, USA

**Keywords:** culture and social determinants of health, autism screening, complex attention deficit hyperactivity disorder (adhd), delayed diagnosis, autism spectrum disorder (asd)

## Abstract

Autism spectrum disorder (ASD) presents a diagnostic challenge due to its highly heterogeneous nature. The most common clinical manifestations include difficulty with social interaction and the presence of repetitive sensory-motor behaviors. Females are more likely to be misdiagnosed or have a delayed diagnosis compared to males. Other factors that contribute to delayed diagnosis include low socioeconomic status and belonging to an ethnic minority. In pediatrics, the goal of ASD screening is to diagnose ASD earlier, with timely referral to early intervention services, so that better long-term neurodevelopmental outcomes can be achieved. Moreover, attention deficit hyperactivity disorder (ADHD) is the most common comorbidity in patients with ASD. While the Diagnostic and Statistical Manual of Mental Disorders fourth edition (DSM-4) prohibited a co-diagnosis of autism and ADHD, the DSM-5 has modified exclusion criteria to allow such. This case describes a minority adolescent male patient who presented initially with complex ADHD, who upon extensive evaluation, was ultimately diagnosed with co-existing autism. This patient’s diagnosis of ASD at the age of 14 in the setting of a pre-existing complex ADHD diagnosis demonstrates how symptoms of inattention or hyperactivity may convolute underlying or newly emerging social interaction difficulties. We highlight how children who are diagnosed with ADHD should be screened or evaluated for autism in the right clinical setting, such as evident persistence of social interaction impairment despite ADHD treatment.

## Introduction

Autism spectrum disorder (ASD) is a highly heterogeneous disorder that presents with persistent difficulties with communication and social interaction, in addition to restrictive, repetitive, or stereotyped sensory-motor behaviors [1,2]. ASD has been noted as a shared occurrence in all racial, ethnic, and socioeconomic groups, although children from minority groups are less consistently diagnosed with ASD compared to Caucasian children [1,2]. According to the World Health Organization, one in 160 children internationally has a diagnosis of ASD. However, the prevalence of ASD in low- and middle-income countries is unknown [3-5]. Approximately one in 44 children have been diagnosed with ASD in the US, according to the Centers for Disease Control and Prevention (CDC) Autism and Developmental Disabilities Monitoring (ADDM) Network [5]. ASD is more common in males, with the male-to-female ratio estimated to be three-to-one [2,6]. Stereotyping of ASD as a male disorder has been a contributing factor to females being more likely to be misdiagnosed or having delayed diagnosis [2,7].

Poor eye contact, decreased responsiveness to name, inability to show and share, and absence of gesturing by 12 months are concerning for early signs of ASD [2]. Screening for younger children includes the Modified Checklist for Autism in Toddlers, Revised, with Follow-up (M-CHAT-R/F) and Survey of Wellbeing of Young Children (SWYC) [2,8-9]. Older children may demonstrate struggles with figurative thinking, understanding emotions, and conversational skills. The Autism Spectrum Screening Questionnaire (ASSQ), Social Responsiveness Scale (SRS), and Social Communication Questionnaire (SCQ) are available for the screening of older children that display signs of autism [2,10-11]. The Autism Diagnostic Observation Schedule second edition (ADOS-2) is a gold standard tool to assess social interaction and communication in the assessment of ASD. The American Psychiatric Association’s Diagnostic and Statistical Manual of Mental Disorders fifth edition (DSM-5) criteria was published in 2013 with the intention of making the diagnosis of ASD more straightforward and allowed the diagnoses of ASD to be made with co-occurring psychiatric conditions, such as attention deficit hyperactivity disorder (ADHD) [1].

The literature has shown that Early Intensive Behavioral Intervention (EIBI) is beneficial for some children with ASD, improving overall outcomes later in life [12]. This case characterizes a 14-year-old minority male patient who presented initially with complex ADHD and a diagnosis of bipolar II disorder, who, upon comprehensive psychologic and psychiatric evaluation, was ultimately diagnosed with ASD with complex ADHD. 

## Case presentation

At the age of 13, the patient established care with our pediatrics clinic for his well-child exam. The patient’s mother voiced concern regarding longstanding difficulty with “making and keeping friends” that had worsened since the age of nine in the setting of a complicated psychiatric history.

The patient had been diagnosed at the age of 12 with complex ADHD in the setting of bipolar II disorder by an outside psychiatric provider prior to establishing care with our clinic. He was started on methylphenidate and aripiprazole. The patient’s mother noted improved mood, affect, and decreased difficulty making friends after starting medical treatment. The mother reported that another psychiatric provider, whose notes were not disclosed to us, noted a lack of classic symptoms of ADHD, given that the patient was an excellent student with no problems of inattention, hyperactivity, or behavioral concerns. This provider recommended that methylphenidate be discontinued due to concern for appetite suppression. The patient’s mother noted worsening impaired social interaction and declining grades following the discontinuation of methylphenidate, which prompted the medication to be restarted by our clinic. 

During the first encounter with our clinic, the patient was noted to have poor eye contact. The patient was described by his mother to have struggled with engaging with same-age peers and frequent avoidance of social activities, even while on methylphenidate. Gestational and developmental histories were unremarkable, with no developmental delay for motor and speech milestones noted. The patient was noted to lack functional play and would only engage in functional play if modeled by his mother. The patient also did not engage in mimicking or playing telephone. Family history amongst first-degree relatives was remarkable for suicide attempts without completion, depression, anxiety, bipolar disorder, and ADHD. The parent and patient agreed to have the evaluation for autism scheduled due to his persistent social interaction impairment. 

A comprehensive psychological evaluation by a clinical psychologist revealed that although the patient had a history of having a few friends, he reported that he struggled to recognize if someone was his friend. He specifically struggled with interpreting nonverbal forms of communication, such as interpreting body language. Per his mother, he demonstrated a preference for engaging in activities independently outside of school. The patient reported that he could recognize emotions in others but lacked an understanding of how to respond to these emotions. The patient’s affect was flat with a restricted range. He struggled in sustaining conversation and providing insight into his emotional experiences. The patient was found to have sensory sensitivities to clothing and foods. Several tests were administered, including the Stanford Binet fifth edition (SB-5), Autism Diagnostic Observation Schedule-2 (ADOS-2) - module 4, and Delis-Kaplan Executive Function System (D-KEFS) tests. Cognitive abilities fell in the high average range, with a full-scale IQ (FSIQ) of 113 (81st percentile). The patient was found to have high evidence of autism-related symptoms on the ADOS-2, which in conjunction with his clinical evaluation, indicated that he met the criteria for the diagnosis of ASD level 1. The patient’s D-KEFS test suggested that the patient’s occasional difficulties with attention and executive function were not consistent with a diagnosis of ADHD but were relative to perseverative thought and slower processing connected with the primary diagnosis of ASD. However, methylphenidate was continued due to functional response to the medication. Furthermore, psychiatry re-evaluated the patient and noted that, regardless of the D-KEFS test results, he still met DSM-5 criteria for ADHD. The patient currently continues to follow with outpatient psychiatry and is currently on amphetamine/dextroamphetamine. His symptoms were further clarified, and it was determined that there was no history of mania or hypomania that would be consistent with a diagnosis of bipolar disorder. The diagnosis of bipolar II disorder was removed, and aripiprazole was discontinued. The depressive features noted in his initial diagnosis of bipolar II disorder were also re-evaluated, and it was determined that he had a diagnosis of major depressive disorder (MDD) instead. Therefore, escitalopram was initiated in place of aripiprazole. His ultimate diagnosis is ASD with co-existing complex ADHD in the setting of MDD.

## Discussion

Early ASD diagnosis and individualized intervention plans have been associated with better overall outcomes than when receiving a delayed diagnosis [13,14]. Autism symptom severity, socioeconomic factors, and the presence of psychiatric disorders at baseline have been shown in the literature to be associated with delayed diagnosis of ASD [14].

ADHD is the most common comorbidity in people with ASD, with rates of co-occurrence cited in the literature ranging from 25% to 81% [15]. Overlapping symptomatology of ASD and ADHD include deficits in social communication and attention. While these deficits are more commonly attributed as features of ASD, research has shown that children with ADHD can demonstrate ASD traits, including difficulties in forming relationships, developing awareness of the feelings of others, and having impaired nonverbal communication [16,17]. Similarly, features of ADHD symptoms, such as inattention and hyperactivity, can present in individuals with ASD without meeting the diagnostic criteria of ADHD [16]. While DSM-4 prohibited a co-diagnosis of autism and ADHD, the DSM-5 modified the exclusion criteria to allow such, as more studies have demonstrated shared traits between the two disorders. Overlapping symptomatology and the high frequency of ADHD symptoms in autism can lead to a misdiagnosis or delayed ASD diagnosis, as demonstrated by this case. In a study of 2212 individuals with ASD who were controlled for ASD severity and age, male children and adolescents with a pre-existing ADHD diagnosis received an average delay of 1.5 years in obtaining a diagnosis of autism, relative to those without a prior diagnosis of ADHD [16].

This patient’s diagnosis of ASD at the age of 14 in the setting of a pre-existing complex ADHD diagnosis demonstrates how symptoms of inattention or hyperactivity may convolute underlying or newly emerging social interaction difficulties. Considering the growing body of research surrounding the association of ADHD and ASD, children who are diagnosed with ADHD should be screened or evaluated for autism in the right clinical setting, such as evident persistence of social interaction impairment despite ADHD treatment. Methylphenidate and atomoxetine are medications commonly used in the treatment of ADHD and have been noted to demonstrate a varying level of effectiveness in children with co-occurring ASD and ADHD. There is evidence in the literature that suggests medication response rates are markedly lower in children with ASD than in children without ASD. Also, individuals with co-occurring ASD and ADHD have been shown to have an increased risk of experiencing side effects [18]. The patient presented in this case demonstrated improved yet persistent social difficulties despite methylphenidate treatment, which directed autism screening and evaluation.

While a pre-existing diagnosis of complex ADHD was primarily attributed as the underlying factor for delayed diagnosis of ASD in the male adolescent patient for this case, other factors are also contributory. Delayed or missed diagnoses of ASD have been shown in the primary literature to be associated with patients belonging to an ethnic minority, lower parental education level, and low socioeconomic status [14]. All of these listed factors are present in this case. Lack of healthcare access, cultural stigma, and language barriers are thought to contribute to this healthcare discrepancy amongst ethnic minorities and lead to delayed diagnosis [2]. The patient presented to a Medicaid-only clinic in our state after having moved several times before establishing care at this clinic. This resulted in a lack of continuity of care that also contributed to the protracted course leading to his diagnosis of ASD as an adolescent. 

## Conclusions

ASD is a highly heterogeneous disorder characterized by social communication deficits and the presence of restricted interests and repetitive behaviors that continues to serve as a diagnostic challenge. ASD core symptoms can be confounded by other neurodevelopmental disorders and comorbidities, especially in cases of a pre-existing diagnosis of ADHD. Screening for ASD in children diagnosed with ADHD should be considered in the setting of persistent social impairment or newly emerging social difficulties despite ADHD treatment. Socioeconomic and cultural factors have also been frequently cited as contributory factors to delayed diagnosis of ASD and should be considered when considering ASD screening and evaluation. Early diagnosis allows for early treatment with individualized intervention and access to public support systems, which ultimately results in better overall outcomes for children than when diagnosed at later ages. Children with a delayed diagnosis of ASD will still likely benefit from interventional services.
